# Evaluation of pro-inflammatory cytokines in frail Tunisian older adults

**DOI:** 10.1371/journal.pone.0242152

**Published:** 2020-11-09

**Authors:** Sonia Hammami, Imen Ghzaiel, Souha Hammouda, Nabil Sakly, Mohamed Hammami, Amira Zarrouk

**Affiliations:** 1 Biochemistry Laboratory, LR12ES05 LR-NAFS ‘Nutrition - Functional Food & Health’ Faculty of Medicine of Monastir, Monastir, Tunisia; 2 Department of Internal Medicine, CHU F Bourguiba Monastir, Geriatric Unit, Monastir, Tunisia; 3 Faculty of Sciences of Tunis, University of Tunis El Manar, Tunis, Tunisia; 4 Laboratory of Microbiology, Unity of Immunology of EPS Fattouma Bourguiba, Monastir, Tunisia; 5 Biochemistry Laboratory, Faculty of Medicine of Sousse, Sousse, Tunisia; University of Houston, UNITED STATES

## Abstract

The present study was undertaken to evaluate serum levels of pro-inflammatory cytokines in Tunisian older adults and to examine the relationships between inflammatory marker levels, geriatric, and biochemical parameters. A cross-sectional study was conducted in a population of Tunisian older adults (N = 141, aged 65 and over). Patients were recruited from the Department of Internal Medicine, Fattouma Bourguiba University Hospital (Monastir, Tunisia) and from a nursing home (Sousse, Tunisia). Comprehensive geriatric assessment, history taking and examination including functional and nutritional assessment were done for each participant. Enzyme-linked immunosorbent assay (ELISA) test was used to measure serum cytokine (TNF-α, IL-8, IL-6) levels. The modified Short Emergency Geriatric Assessment score (SEGAm) were used to classify patients as 51 very-frail, 40 frail, and 50 non-frail. The age of the participants (80 men, 61 women) ranged from 65 to 97 years. Serum levels of TNF-α, IL-8 and C-reactive protein (CRP) were significantly higher in very-frail participants compared to frail and non-frail ones. However, no significant differences in IL-6 levels were detected among frailty groups. After adjustment for age, CRP and IL-8 levels remained significantly associated with frailty. Analysis of the receiver operating characteristic (ROC) curve corresponding to IL-8 showed an area under the curve of 0.7 (*p* = 0.003; 95% CI [0.58–0.81]) and a predictive threshold of 5.27 pg/ml. Positive correlations were found between frailty score, IL-6, and IL-8 levels. In addition, a significant positive correlation was observed between IL-8 levels and Timed Up and Go test results. However, a negative correlation was observed between Mini Nutritional Assessment Short-Form score, IL-6 and CRP levels, as well as between Activities of Daily Living score and serum levels of TNF-α, IL-6, and CRP. In conclusion, the key findings of this study collectively support a role of pro-inflammatory cytokines, TNF-α, CRP, and especially IL-8 in the development of frailty in older adults.

## Introduction

Aging of the population is a worldwide phenomenon. In Tunisia, the proportion of older people aged more than 60 years has grown steadily since 1966, increasing from 8% in 1995 to 12.5%, and is anticipated to reach 25% in 2050 [1, 2]. Faced with an increasingly older population, clinicians find it useful to identify geriatric conditions in order to determine a frail person’s medical, psychological, and functional capability [3]. Frailty is a multidimensional geriatric syndrome characterized by a state of age-related biological vulnerability to stressors, and decreased physiological reserves associated with alterations in energy metabolism, decreased skeletal muscle strength and mass, and dysregulation of inflammatory cytokines [4]. Among these factors, inflammation has been postulated as a major area of interest within the field of frailty in older people [5].

It is widely accepted that the immune system undergoes quantitative and qualitative modifications during aging [6]. This phenomenon, called immunosenescence, is accompanied by an increase in pro-inflammatory cytokines and a reduction in anti- inflammatory cytokines, leading to a chronic low-grade inflammatory state [5, 7] which might contribute to frailty [8]. Previous studies have shown that in older adults, higher circulating levels of tumor necrosis factor-α (TNF-α), interleukin-6 (IL-6) and C-reactive protein (CRP) are associated with decreased muscle strength, poor physical function, frailty, and mortality [9]. Serum levels of TNF-α were also strongly associated with nutritional status in elderly women [10]. Higher serum IL-6 levels were seen in older people with deficits in activities of daily living performance, mobility, and sarcopenia [11]. Furthermore, higher levels of CRP were associated with increased risk of mortality in frail older adults, but they were not considered a significant predictor of mortality [12]. In light of the growing body of published research about elevated markers of inflammation such as TNF-α, IL-6, and CRP in frailty, attention has recently turned to the relationship between IL-8, pro-inflammatory cytokines, and the geriatric status and severity of frailty in older people.

To date, it is unclear whether IL-8 plays an important role in frailty. Therefore, the main aim of the present study was to investigate the association between pro-inflammatory markers and the development of frailty. First, inflammatory markers related to different frailty stages in older people were studied. Second, the relationship between inflammatory markers, geriatric and biochemical parameters in frail patients was explored. Finally, a potential cut-off point for inflammatory biomarkers was determined and tested as a predictor of frailty in our participants.

## Materials and methods

### Patients

This cross-sectional study conducted in Tunisia enrolled 141 older adults aged 65 years and more (80 men, 61 women) over a period of 1 year from March 2018 to March 2019. All participants were recruited from the Department of Internal Medicine, Fattouma Bourguiba University Hospital (Monastir, Tunisia) and from a nursing home (Sousse, Tunisia). Patients who were younger than 65 years, unable to communicate, who had major medical urgencies or severe dementia, or who did not provide their informed consent were excluded.

Data were collected through questionnaires, medical history, clinical examination, geriatric assessment, and laboratory analysis. Weight and height were measured using standard techniques. Body mass index (BMI) was calculated as weight (kg) divided by height (m) squared. The frailty status of our patients was evaluated using the modified version of the Short Emergency Geriatric Assessment (SEGAm) validated in 2014 by the French Society of Geriatrics and Gerontology [13]. The SEGAm includes the parameters age, patient provenance, number of medicines, cognitive functions, humor, nutritional status, degree of dependency, history of falls, and comorbidities. Each item was scored as 0 (most favorable state), 1, or 2 (least favorable state). The maximum score is 26 points, representing the highest level of frailty. Individuals scoring from 0 to 8 points are considered “non-frail”, scores of 9 to 11 are considered “frail”, and scores of 12 points or more “severe frail”. All patients underwent comprehensive geriatric assessment (CGA) including an evaluation of depression (Mini Geriatric Depression Scale [Mini-GDS]) [14], cognitive status (Mini-cognitive [Mini-Cog]) [15], nutritional status (Mini Nutritional Assessment Short-Form [MNA-SF] with a cut-off score of 8) [16], functional status (Activities of Daily living [ADL]) [17], and gait performance and mobility (Timed Up and Go test [TUG]) [18]. Activities of daily living were assessed with the Katz score, which comprises six items (bathing, dressing, toileting, transferring, continence and feeding); each item was scored as dependent *vs* independent. The total score ranges from 0 (dependent) to 6 (independent). Dependency was defined as deterioration in at least one domain of the ADL (score < 6). The CGA was considered abnormal if the patient had one of the following criteria: Mini-GDS ≥ 1/4, MNA-SF ≤ 8/14, ADL ≤ 5/6, or TUG ≥ 20 seconds.

Ethical approval for the study was granted by the Research Ethics Committee of Fattouma Bourguiba University Hospital, and written informed consent was obtained from all participants. The CGA and data collection were carried out by a trained clinical research associate.

### Biomarker measurements

Venous blood samples were collected into serum tubes after overnight fasting. All samples were centrifuged at 3500 rpm for 10 min at 4°C, and the serum was divided into several aliquots which were immediately frozen at −80°C and stored until pro-inflammatory cytokine (TNF-α, IL-6, and IL-8) analysis. Serum pro-inflammatory markers were detected by sandwich enzyme‑linked immunosorbent assay (ELISA), and all assays were performed according to the manufacture’s protocols. Conventional biochemical parameters in older patients, including CRP, glycemia, glycated hemoglobin, and albumin, were measured at the biochemistry laboratory of Fattouma Bourguiba University Hospital in Monastir.

### Statistical analyses

General results in the study population, classified according to frailty status, were analyzed with the Statistical Package for Social Sciences (SPSS 16.0 for Windows). The distribution of variables was checked for normality with the Kolmogorov–Smirnov test. Clinical, geriatric, and biochemical characteristics of the participants with different frailty status were compared with the χ^2^ test for categorical variables, parametric one-way ANOVA, and Duncan’s multiple range test or non-parametric Kruskal–Wallis test for continuous variables. The Spearman correlation test was used to determine the relationships between inflammatory biomarkers, geriatric, and biochemical parameters among frailty groups. Univariate and age adjusted analyses were conducted. The results were considered statistically different at a *p*-value of 0.05 or less. Receiver operating characteristic (ROC) curves were used to test the ability of the inflammatory biomarkers to discriminate non-frail from frail participants.

## Results

### Clinical, geriatric and biochemical characteristics of patients

The patients were classified according to their SEGAm score into three groups as follows: group 1, non-frail (SEGAm ≤8, n = 50 patients); group 2, frail (8 < SEGAm ≤11, n = 40 patients); and group 3, very-fail (SEGAm >11, n = 51 patients). The characteristics of this study population are summarized in Table 1. Very-frail participants were significantly older than frail and non-frail participants, with a median age of 80 [range 73–85] years *vs* 77 [71–81.7] years in the frail group, and 69 [66–72.2] years in the non-frail group (*p*<0.001). The majority of frail and very-frail participants were living in nursing homes, while more than 80% of non-frail participants were living with their family. Very-frail participants, compared to frail and non-frail ones, had a significantly lower BMI of 23.27±4.59 kg/m^2^*vs* 25.92±4.92 kg/m^2^ and 26.11±4.71 kg/m^2^ for very frail, frail and non-frail counterparts, respectively (*p* = 0.014). The Mini-Cog score decreased with frailty severity. Only 36% of non-frail individuals were at risk of depression *vs* more than 90% of very-frail patients. The MNA-SF score decreased, and consequently the risk of malnutrition increased with frailty severity. Severely frail patients had the lowest ADL score (*p*<0.001). Only 16% of the non-frail group had ADL dependency, whereas ADL dependency was present in 96% of the very-frail group. The TUG test times increased significantly with frailty severity. Mean serum albumin level was significantly lower in the very-frail group compared to the frail and non-frail groups. Glycemia decreased significantly as frailty increased.

**Table 1:** Characteristics of older adults according to SEGAm score.

Characteristic	Non-frail n = 50	Frail n = 40	Very-frail n = 51	*p* value
**Assessment of general data**				
Age, years, median (range)	69 (66–72.25)	77 (71–81.75)	80 (73–85)	**<0.001**
Age ≥ 80 years, n (%)	4 (8)	15 (37.5)	26 (51)	**<0.001**
Gender: female/male, n (%)	15/35	19/21	27/24	0.054
BMI, kg/m^2^, mean ± SD	26.11 ± 4.71 ^a^	25.92 ± 4.92 ^a^	23.27 ± 4.59 ^b^	**0.014**
**Assessment of living condition**				**<0.001**
With spouse, n (%)	30 (60)	6 (15)	9 (17.6)	
With children, n (%)	12 (24)	9 (22.5)	12 (23.5)	
Nursing home, n (%)	8 (16)	25 (62.5)	30 (59)	
**Assessment of general health status**				
Diabetes, n (%)	24 (48)	10 (25)	17 (33.3)	0.068
Hypertension, n (%)	22 (44)	20 (50)	30 (58.8)	0.325
Dyslipoproteinemia, n (%)	7 (14)	5 (12.5)	5 (9.8)	0.807
Other, n (%)	32 (64)	28 (70)	43 (84.3)	0.062
Polypharmacy ≥ 3 n (%)	30 (60)	23 (57.5)	35 (69)	0.503
**Assessment of cognitive status and mood**				
Mini-Cog score, median (range)	4 (3–5)	2 (2–3)	1 (0–2)	**<0.001**
Mini-GDS score, median (range)	0 (0–1)	3 (1–4)	4 (4–4)	**<0.001**
Mini-GDS ≥ 1, n (%)	18 (36)	33 (82.5)	46 (90.2)	**<0.001**
**Assessment of nutritional status**				
MNA-SF score, median (range)	12 (10–14)	8.5 (7–10)	5 (4–7)	**<0.001**
MNA-SF at risk of malnutrition ≤ 8, n (%)	2(4)	12 (30)	44 (86.3)	**<0.001**
**Assessment of living independence**				
ADL score, median (range)	6 (3.5–6)	4.5 (3.6–5.5)	2 (0–4.5)	**<0.001**
ADL ≤ 5, n (%)	8 (16)	22 (55)	49 (96)	**<0.001**
**Assessment of physical performance**				
Timing TUG, seconds, median (range)	17.23 (13.76–20.46)	27.41 (18.23–29.73)	28.49 (20.81–31.52)	**<0.001**
**Assessment of biochemical data**				
Glycemia, mmol/l, median (range)	7.3 (5.8–10.2)	5.9 (4.9–6.55)	5.5 (4.6–7.8)	**0.014**
HbA1c, %, median (range)	7.7 (6.8–8.6)	6.4 (5.7–8)	6.9 (6.1–9.32)	0.36
Albumin, g/l, mean ± SD	36.30 ± 6.16 ^a^^,^^b^	39.81 ± 4.69 ^a^	33.65 ± 9.66 ^b^	**0.006**

^a, b^ Different superscript letters in the same row indicate significant difference between values at *p*<0.05 (Duncan’s multiple range test).

BMI, Body mass index; Mini-Cog, Mini Cognitive; Mini-GDS, Mini Geriatric Depression Scale; MNA-SF Mini Nutritional Assessment Short-Form ADL, Activities of Daily Living; TUG, Timed Up and Go; HbA1c, hemoglobin A1c.

### Serum levels of inflammatory biomarkers

The inflammatory biomarker levels tested in all three frailty groups are shown in Table 2. TNF-α level was significantly higher in the very-frail group (28.71 pg/ml [27–32.78]) than in the frail (25.54 pg/ml [24.23–28.03]) and non-frail groups *vs* (21.16 pg/ml [25.33–30.97]) (*p<*0.003). Similarly, IL-8 levels were significantly higher in the very-frail group (22.31 pg/ml [12.63–32.98]) compared to the frail (9.69 pg/ml [6.66–17.95]) and non-frail groups (19.91 pg/ml [11.34–24.95]) (*p<*0.001). The very-frail group had also a significantly higher CRP level compared to the other patients. As expected, serum CRP levels showed a strong, significant association with prevalent frailty after adjusting for age. Likewise, IL-8 was associated with frailty in the unadjusted and age-adjusted models. The association of TNF-α with frailty reached borderline statistical significance after adjusting for age. As in the unadjusted analysis, there was no age-adjusted association between IL-6 level and frailty group.

**Table 2:** Baseline inflammatory biomarker profile.

Inflammatory biomarkers	Non-frail	Frail	Very-frail	Unadjusted	Adjusted
	n = 50	n = 40	n = 51	*p* value	*p* value
TNF-α, pg/ml,	21.16	25.54	28.71	**0.003**	**0.046**
median (range)	(25.33–30.97)	(24.23–28.03)	(27–32.78)		
IL-8, pg/ml,	19.91	9.69	22.31	**<0.001**	**0.033**
median (range)	(11.34–24.95)	(6.66–17.95)	(12.63–32.98)		
IL-6, pg/ml,	12.22	9.19	11.08	0.118	0.421
median (range)	(8.03–16.24)	(7.73–11.05)	(8.38–18.52)		
CRP, mg/l,	12.13	6	63	**0.01**	**0.003**
median (range)	(4.97–28.5)	(3.27–60.4)	(12.10–104.5)		

TNF-α, tumor necrosis factor-α; IL-8, interleukin-8; IL-6, interleukin-6; CRP, C-reactive protein.

Adjusted *p* value obtained after adjustment for age.

**Fig 1:**
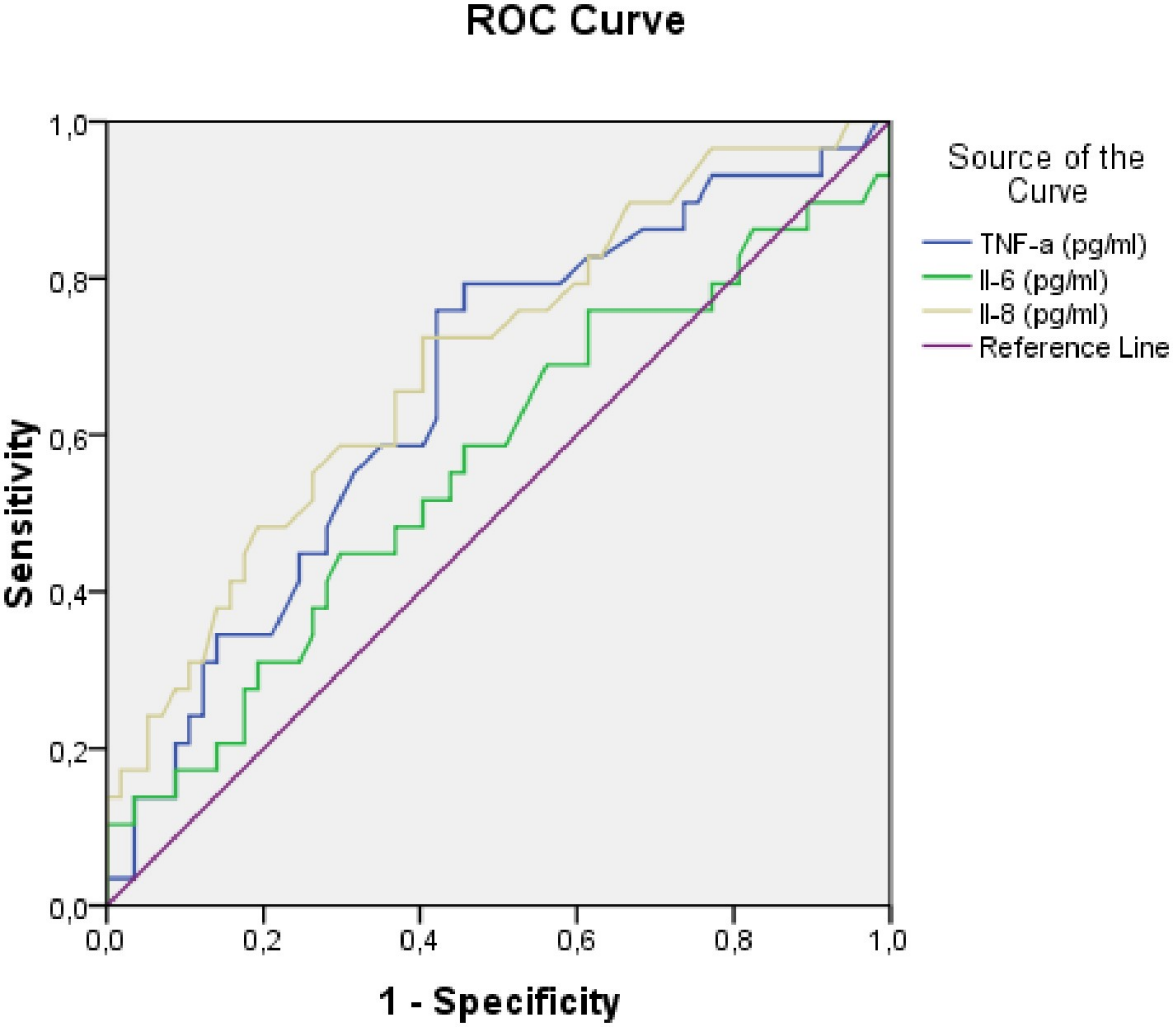
Receiver-operating characteristic (ROC) curves for TNF-α, Il-6 and Il-8 as predictors of frailty. TNF-α, tumor necrosis factor-α; IL-6, interleukin-6; IL-8, interleukin-8.

The ROC curve was performed to assess how well pro-inflammatory biomarkers predicted frailty. The non-frail and frail groups were used as the standard (Fig 1). An IL-8 level of 5.27 pg/ml was considered the predictive threshold for frailty, with an area under the ROC curve (AUC) of 0.7 (*p* = 0.003; 95% CI [0.58–0.81]). In addition, a TNF-α level of 22.71 was considered the predictive threshold of frailty, with an AUC of 0.66 (*p* = 0.016; 95% CI [0.54–0.79]).

### Correlation between inflammatory biomarkers, geriatric parameters, and functional status in frailty groups

Possible correlations between inflammatory biomarkers, geriatric parameters, and functional status were investigated in each frailty group. Frailty score was positively associated with serum levels of the inflammatory biomarkers IL-6 (*r* = 0.34, *p* = 0.001), IL-8 (*r* = 0.282, *p* = 0.007), and CRP; (*r* = 0.33, *p* = 0.005) (Table 3). Evaluation of the relationship of inflammatory biomarkers with nutritional status and functional performance showed that IL-8 levels were associated with poorer physical performance as reflected by higher TUG times (*r* = 0.31, *p* = 0.03). However, IL-6 was negatively correlated with MNA-SF (*r* = −0.25, *p* = 0.02) and ADL scores (*r* = −0.33, *p* = 0.002). In addition, CRP was negatively correlated with MNA-SF (*r* = −0.37; *p* = 0.001) and ADL scores (*r* = −0.38; *p* = 0.001). Albumin level was negatively correlated with TNF-α (*r* = −0.22; *p* = 0.04), IL-6 (*r* = −0.43; *p*<0.01) and CRP level (*r* = −0.62, *p*<0.01). Serum HbA1c was positively correlated with IL-6 level (*r* = 0.51, *p* = 0.005).

**Table 3:** Correlations between inflammatory biomarkers and geriatric and biochemical parameters.

Parameters	TNF-α		IL-6		IL-8		CRP	
	*r*	*p*	*r*	*p*	*r*	*p*	*r*	*p*
**Geriatric parameters**								
Frailty SEGAm score	0.15	0.17	0.34	**0.001**	0.28	**0.007**	0.33	**0.005**
TUG time	0.1	0.52	0.11	0.45	0.31	**0.03**	0.03	0.823
MNA-SF score	−0.10	0.34	−0.25	**0.021**	−0.15	0.16	−0.37	**0.001**
ADL score	−0.218	**0.04**	−0.33	**0.002**	−0.21	**0.05**	−0.38	**0.001**
**Biochemical parameters**								
Albumin, g/l	−0.22	**0.04**	−0.43	**<0.001**	0.05	0.65	−0.62	**<0.001**
HbA1c, %	−0.13	0.50	0.51	**0.005**	0.38	**0.047**	0.35	0.13

TNF-α, tumor necrosis factor-α; IL-8, interleukin-8; IL-6, interleukin-6; CRP, C-reactive protein; SEGAm, modified Short Emergency Geriatric Assessment; TUG Timed Up and Go; MNA-SF, Mini Nutritional Assessment Short-Form; ADL, Activities of Daily Living; HbAIc, hemoglobin A1c.

## Discussion

This study demonstrates that, compared to non-frail older persons, those with frailty were more likely to be 80 years old or more, with a predominance of females, those living in nursing home, and those with low BMI, in accordance with other studies of frail elderly populations [19, 20]. In addition, our findings indicate that frailty is mostly associated with cognitive impairment, risk of depression, altered nutritional status, and a decline in functional domains (ADL and TUG). This link between frailty and cognitive, nutritional, and functional decline has also been reported in previous studies [21–24]. Frailty is the most problematic expression of aging. The causes of this syndrome are physical, biological, social, and environmental factors. Thus, frailty is characterized by age-associated cumulative declines in multiple physiological systems, with weakness, low physical activity level, and malnutrition. People categorized as frail were observed to have more adverse outcomes compared to non-frail people, e.g. dependency, disability, falls and delirium [25].

The present study provides a comprehensive examination of the relationships between pro-inflammatory markers (TNF-α, IL-6, IL-8, and CRP) and frailty. Our results show positive associations between frailty score and serum IL-6, IL-8, and CRP levels. Some recent studies have also noted that the severity of frailty is associated with pro-inflammatory state, which has been considered to be at the center of the process leading to frailty [26–28]. It is generally recognized that older age is characterized by increased serum levels of pro-inflammatory markers. This dysregulation suggests a low level of chronic inflammation–a phenomenon called “inflammaging”–which contributes to the pathogenesis of age-related diseases and exacerbation of chronic diseases [29]. Singh et al. reported that aging is accompanied by increases in serum levels of several pro-inflammatory mediators even in apparently healthy individuals, and in the absence of acute infection [30]. This persistent inflammation, also designed low-grade inflammation, is considered a key component in the development of frailty syndrome [29–31]. Bian et al. found that with aging, inflammatory factors increased significantly, and serum pro-inflammatory markers increased two- to four-fold, even in elderly persons without chronic diseases [32]. In contrast, two studies that followed older participants without frailty at baseline, showed that levels of both CRP and IL-6 were not associated with a higher risk of frailty [33, 34]. A possible hypothesis for this lack of association is that these inflammatory markers are not associated with a predisposition to frailty, but may increase once frailty has set in. Another hypothesis is that frail people are sensitive to acute diseases that could increase inflammatory parameters during the follow-up period; however, neither of these studies adjusted their analyses for inherent changes in these markers.

In the present study, as shown in Table 2, the frail group had lower levels of IL-6 and IL-8 than the non-frail and very-frail groups. In addition, the group of frail participants had a lower frequency of type 2 diabetes. Studies focusing in diabetes found that patients with type 2 diabetes had a marked elevation of circulating IL-8 and IL-6 levels [35]. These findings support the hypothesis of the potential role of IL-8 and IL-6 as markers of inflammation in patients with diabetes.

A recent meta-analysis that included 32 cross-sectional studies and 23,910 older participants concluded that frailty was associated with significantly higher serum inflammatory parameters (CRP, IL- 6 and TNF-α) compared to non-frail participants [27]. The relationship between inflammation and frailty appears to be complex and multifactorial. First, as confirmed by our analyses, frail persons are older than robust participants, and as noted above, aging is associated with low-grade inflammation called inflammaging. Second, frail people appear to have a significant decline in immune parameters such as T-cell activity and antibody production, associated with an increase of oxidative stress markers and a decline in antioxidant capacity leading to the accumulation of pro-inflammatory factors. These changes are called immunosenescence and are lifestyle-modifiable factors that may also play a key role in inflammatory processes [5]. Finally, frailty with aging is associated with a decline in muscle mass coupled with an increase in fat mass. In fact, the combination of sarcopenia and obesity (sarcopenic obesity) has a detrimental impact on and important consequences for systemic inflammation [33, 36]. In this connection, it was shown that adipocytes release more reactive oxygen species and pro-inflammatory cytokines, leading to a continuous state of inflammation [37].

Furthermore, the present study demonstrates that very-frail participants had significantly higher IL-8 levels than non-frail and frail ones. Analysis of the ROC curve corresponding to IL-8 showed an AUC of 0.7 (*p* = 0.003; 95% CI [0.58–0.81]) and a predictive threshold of 5.27 pg/ml. This suggests a 70% likelihood of correctly distinguishing a frail from a non-frail patient based on the concentration of IL-8. Lower serum IL-8 level correlated with worse functional status (ADL, TUG), and like serum IL-6 and CRP levels, was positively correlated with frailty scores. Few previous studies have reported higher circulating IL-8 concentration in the frail older population [38, 39]. Recently, Hsu et al. found a weak association between IL-8 and frailty, which suggests that it might also have a role in this syndrome [38]. Edvardsson et al. also reported that elevated levels of IL-8 during 1-year follow-up were related to reduced life expectancy in nursing home residents [39]. The present study is, to the best of our knowledge, the first designed to investigate IL-8 profile in elderly persons with frailty and investigate its relationship with both the findings of geriatric evaluation and biochemical parameters in the Tunisian population. However, the mechanism underlying the association between IL-8 and frailty in older persons remains unclear. Both IL-6 and IL-8 have been previously proposed as important intervening cytokines in cellular senescence and the senescence-associated secretory phenotype (SASP) [38]. In light of previous findings taken together with the positive correlations in the present study, we postulate that these cytokines are not only markers of chronic inflammation, but may also precipitate aging-related frailty.

We also investigated the association between serum inflammatory markers (IL-6, IL-8, TNF-α, and CRP) and overall functional decline (ADL and TUG) and malnutrition. Serum levels of the inflammatory biomarkers TNF-α, IL-6, IL-8, and CRP were negatively correlated with ADL score. Additionally, both IL-6 and CRP levels were negatively correlated with MNA-SF score, along with serum albumin level. This study, in consonance with earlier research, shows that systemic inflammation marked by elevated IL-6, CRP, and TNF-α levels is associated with functional decline and malnutrition, two central components of the frailty syndrome [40]. In this connection, Soysal et al. reported that the MNA can be used to detect frailty with a sensitivity of 66.9% and a specificity of 85.4%, suggesting a very close relationship between nutritional status and frailty [41, 42]. In addition, higher levels of inflammatory markers were associated with greater loss of muscle mass and strength, malnutrition leading to sarcopenia and depression in older adults, all of which are essential elements of frailty [30]. Regarding functional status, a recent prospective study of 88 elderly participants by Ebeid et al. concluded that frailty was predictive of disability [3]. Moreover, a prospective study of 130 older adults by Ma et al. concluded that serum levels of IL-6 were negatively correlated with gait speed, which indicates that inflammation is a molecular mechanism underlying physical frailty [43]. In fact, frailty increases muscle protein catabolism, resulting in sarcopenia and impaired mobility [42]. One novel aspect of the present study was to examine not only conventional inflammatory biomarkers (CRP, IL-6, and TNF-α), but also IL-8 level. Interestingly, we found a slight association between IL-8 level and ADL and TUG scores, which suggests that this cytokine might also have a role in frailty.

Other biochemical parameters have also been investigated as potential indicators of malnutrition and inflammation in the elderly. For example, albumin, the most abundant protein in human serum, has been used as an indicator of malnutrition in elderly people [44]. Consistent with our results, previous work reported a clear relationship between serum albumin concentrations, frailty and systemic inflammation [45]. Indeed, inflammatory states and in particular, high levels of IL-6 and TNF-α, were two of the main factors causing low levels of serum albumin [44]. A study by Sullivan et al. found that inflammation, particularly as reflected by IL-6 and CRP levels, appears to be a powerful determinant of albumin [46]. In this connection, systemic inflammation was found not only to reduce albumin synthesis but also to increase its degradation and promote transcapillary leakage [47]. As in the present study, earlier research demonstrated that nutritional status was closely associated with the degree of frailty [48–50]. Hence, this association supports the presence of link between pro-inflammatory cytokines and nutritional status. Our findings, in accordance with other studies, provide additional evidence supporting the hypothesis that higher serum levels of inflammatory biomarkers may have a negative impact on nutritional status [51]. Recent evidence has linked higher levels of inflammatory biomarkers to an increased risk of cognitive decline, dementia and even depression [52]. However, we found no association between serum levels of IL-6, IL-8, or TNF-α, and scores on the Mini-Cog or Mini-GDS (results not shown). Other studies have obtained contradictory results, which may be due to differences in methodological approaches and outcome measures [52]. In this regard we note that a possible limitation of the present study was the use of short-form tests only (the Mini-Cog and Mini-GDS) in order to rapidly screen our participants. Our study had other potential limitations. First, the relatively small number of patients along with variability in the data may have decreased the statistical power. Second, the observed associations might be influenced by confounders, because of the small sample size. Lastly, we were unable to apply linear logistic regression analysis to adjust for variables such as diabetes, hypertension, chronic disease, and polypharmacy, all of which may have influenced the inflammatory parameters studied here. Despite these limitations, this study also has some strengths. Detailed information on cognition state, mood, nutritional status, living independence, and physical performance allowed us to better understand the frailty phenomenon in older adults, and the associations of these factors with the inflammatory process. Furthermore, ours is the first study designed to explore the relationship between serum levels of four inflammatory markers and frailty in a sample of North African and Tunisian older adults, who belong to a population known for its high adherence to the Mediterranean diet, which is enriched in nutrients with anti-inflammatory effects. Potentially fruitful areas for further research aimed at elucidating the role of inflammatory mediators in the pathogenesis of frailty, thus include evaluating the primarily causal or compensatory nature of the association between frailty and inflammation, determining whether an association exists between inflammatory markers and nutrient intake, and investigating the possible link between genotype and the development of frailty.

## Conclusion

In conclusion, our findings in a sample of older adults in Tunisia suggest that frailty was most clearly associated with cognitive impairment, risk of depression, altered nutritional status, and the domain of functional decline. In addition frailty was associated with more inflammation since frail participants had significantly higher serum levels of CRP, TNF-α, and IL-8. On the other hand, we found a significant negative correlation between inflammatory markers (IL-6 and CRP) and MNA-SF score. We suggest that increased serum levels of TNF-α, IL-6, IL-8, and CRP may be related with significant detrimental effects on daily living activities. Future work is required to better understand whether these cytokines can be used as potential biomarkers of frailty.

## Supporting information

S1 Data Cytokine data. (XLSX)
